# A national peatland database for the Republic of Ireland

**DOI:** 10.1016/j.dib.2026.112965

**Published:** 2026-06-13

**Authors:** Kilian Walz, David Wilson, Kenneth A. Byrne, Florence Renou-Wilson

**Affiliations:** a Department of Biological Sciences & Bernal Institute, Faculty of Science and Engineering, University of Limerick, Ireland; b Earthy Matters Environmental Consultants, Glenvar, Co. Donegal, Ireland; c School of Biology and Environmental Science, University College Dublin, Belfield, Dublin 4, Ireland

**Keywords:** Organic soil, Histosol, Peatlands, Peat soil, Edaphic properties, Water table, Peatland sampling

## Abstract

This article presents a national peatland database for the Republic of Ireland, covering not only undrained, near-intact bogs with absent land use and management at present but also peatlands that have been reclaimed for grassland, forestry and peat extraction (industrial and domestic) land uses. It presents peat substrate and vegetation data across the entire peat depth for peatland types raised bog (RB), lowland blanket bog (LLBB) and mountain blanket bog (MBB), the major land uses and most prevalent management types (a combination of drainage level and land use/grazing intensity) on these peatland types. It excludes ‘near-intact’ fens, which represent <2% of the total peatland area.

Several key edaphic and vegetation properties, peatland classification information, physical features such as topography, elevation, and land use and management information are included in the database which consists of three major components: a ‘Peatland Dataset’ consisting of detailed site, non-composite soil profile and vegetation data, a ‘CHNSO Dataset’, containing results from elemental analysis of composite samples of the Peatland Dataset samples, and a collection of eight water table measurement records over the period November 2017 to January 2020.

This new database fills a gap by integrating peat properties from the entire peat profile with a representative sampling design. It offers a valuable resource for peatland monitoring programs as well as planning and implementing sustainable management across Irish peatlands through restoration, rewetting and *paludiculture* ('wet agriculture') activities.

Specifications Table

**Table:** 

Subject	Earth & Environmental Sciences
Specific subject area	Edaphic, hydrologic and vegetation properties of peatlands for mapping of peatland degradation status, carbon and nitrogen stocks to support monitoring of peatland degradation and greenhouse gases.
Type of data	Table, Raw, Analysed, Processed
Data collection	Peatland sampling campaign (May 2017 to July 2019) across Republic of Ireland to ascertain a wide range of peatland properties. Multi-stage stratified sampling with three stages, 50 primary units sampled across prevalent peatland land use categories (near-intact, grassland, forestry, peat extraction (industrial and domestic) and management options (deep drained, shallow drained, rewetted). Physical landscape features determined using a list of criteria. Vegetation mapping by vegetation relevées, soil sampling with ‘Russian’ peat sampler. Laboratory analysis of disturbed and non-disturbed samples for 15 peat properties. Peat depth measured with peat sampler supported by peat depth probe. Continuous data loggers in eight installed dip wells over a period of 2 years supported by manual measurements for water level measurements (November 2017 to January 2020).
Data source location	Republic of Ireland; The data is stored in University College Dublin and in University of Limerick as well as the Irish Environmental Protection Agency (EPA).
Data accessibility	Repository name: A national peatland database for the Republic of Ireland (AUGER project) [1] Data identification number: https://doi.org/10.5281/zenodo.16746479 Direct URL to data: https://doi.org/10.5281/zenodo.16746479 Instructions for accessing these data: none
Related research article	Several manuscripts are in preparation by the authors that draw from this database, and the data has already been used in a previous article providing detailed records of SOC content and dry bulk density data for afforested soils in the Republic of Ireland [2]

## 1 Value of the Data

• **Why are these data valuable**?
  The data supports further research into the relationship between peat properties and greenhouse gas emissions. It serves as a basis for a national peat monitoring system to continuously monitor the degradation status of peat, effects of a change in management on peat properties and the change of soil properties on greenhouse gases. With this purpose in mind, several of the sampled sampling plots overlap with sites where greenhouse gas emission data was measured over multi-year periods in the past.
  The datab is valuable for ongoing efforts to conserve and restore peatlands across the Republic of Ireland. It provides data that can be used to identify different land use and management effects on peat, thus providing a good basis for decision making for peatland restoration.
  The sampled peatland sites (n = 50) are valuable for complementary or new studies in related fields such as detailed botanical studies or palaeoecological research. The existing data on peat soil properties and vegetation data, as well as information on site characteristics, help augment such additional research. In this sense, this peatland database can be used as a baseline or reference for future ecological studies.
• **How can these data be reused by other researchers?**
  The edaphic peat data can be used by researchers to expand their investigation into the heterogeneity of peat soils under various land uses and to elucidate how to manage peat soils sustainably. The data can be most useful for researchers and governmental agencies reporting to the UNFCCC to model greenhouse gas fluxes based on edaphic and vegetation properties. It also serves for developing, calibrating and validating digital soil modelling approaches used to predict water level fluctuations and GHG fluxes based on remote sensing-derived environmental covariates and measured field data.
  The data from hydrological measurements is useful to analyze inter-annual and inter-seasonal variations between four different land uses of the two major peatland types raised bog and lowland blanket bog. Moreover, the existing 2-year measurement series of the two peatland sites can be used to assess long-term changes in peatland hydrology for 8 peatland type-land use combinations by comparing an additional measurement series conducted at the same measurement locations after a specified period of time. Thus, the hydrological measurements lay the foundation for long-term monitoring of peatland hydrology of raised and blanket bogs in Ireland.

## 2 Background

Peatlands are important carbon and nitrogen stores, and regulate the climate and water flows in landscapes, regions and globally. Edaphic properties of peatlands are controls of biogeochemical processes, which makes their quantification indispensable for accurately quantifying greenhouse gas emissions and peatland degradation status. Peat soils cover 21–23% of Ireland’s land area, equivalent to 1.4–1.6 million hectares [3,4]. While peat carbon was mapped previously for the entirety of Europe [5], edaphic data for Ireland exists only on a coarse spatial resolution (e.g. LUCAS) or as peat profile descriptions from isolated sites [6,7]. A comprehensive database compiling edaphic, vegetation and peatland landscape features, representative for the Republic of Ireland, has been missing to date.

This database was established as part of a national research project, investigating the relationship of peatland properties with greenhouse gas emissions and removals (AUGER project) and was established to complement previous research on GHG fluxes and existing peat profile information. Importantly, it fills an existing gap by providing data on peat properties from the entire peat profile across the major peatland types raised bog (RB), lowland blanket bog (LLBB) and mountain blanket bog (MBB). The database offers a valuable resource for monitoring of peatland health, including water, carbon and nitrogen, informs GHG modelling, and contributes to planning and implementing sustainable peatland management activities across Irish peatlands including restoration, rewetting and *paludiculture* ('wet agriculture') activities. It provides a good baseline for the continuous monitoring of change in peatland condition over time and can potentially be improved in spatial resolution.

## 3 Data Description

### 3.1 Data repository

The data repository can be accessed via this URL: https://doi.org/10.5281/zenodo.16746479. The repository contains one Excel file (.xlsx) with the peatland property data (‘PeatlandDatabase_ROI’) and 8 other Excel files (.xlsx), one dataset for each of 8 sites where water table was continuously monitored.

### 3.2 Peatland database

The Excel file of the peatland database (’PeatlandDatabase_ROI’) is structured as follows. The Excel workbook contains 4 worksheets, each named differently. The work sheet ‘PeatlandProperties’ contains a data table with 84 variables and 2012 observations of site information and non-composited vegetation and peat samples. These variables are described in the second worksheet ‘MetadataProperties’ (Table 1), with information given about each variable’s name (*ColumnName*), type (*VariableType*), explanatory description (*Description*), and information about their ranges of values (if interval variable) or the acronyms used for levels (if categorial variable) (*Specifications*). An additional column (not shown in Table 1) provides the full names of the acronyms (*FullNames*).

**Table 1:** Metadata of the worksheet ‘PeatlandProperties’ with 84 variables. The column with full names of acronyms is missing in this table; ‘ColumnName’ are the variable names.

ColumnName	VariableType	Description	Ranges or Acronyms
ID	integer	unique identifier of sample	1:2012
PointID	integer	unique identifier of sampling point	1:283
PointInSite	integer	sampling point within sampling site	1:6
Y	numeric	latitude	num values
X	numeric	longitude	num values
Elevation(masl)	numeric	elavation in meter over (mean) sea level	num values
PUID	integer	unique identifier of primary unit	1:12
PU	integer	primary unit within peatland type	1:4
PUname	character	name of primary unit	``char values''
PLT	character	peatland type	``char values''
SiteID	integer	unique identifier of sampling site	1:50
SiteName	character	name of sampling site	``text''
LUC	character	land use category	``nat'',``for'',``gra'',``extdom'',``extind''
Management	character	management	``undr'',``dr'',``rw''
MacroTopography	character	topography on landscape level	``FL'',``GS'',``SS'',``D'',``HT''
StructuralChanges	character	topographical and structural changes to site	``bb'',``ss'',``lc'',``sc'',``ge",``re'',``ad'',``opp'',``pp''
LUCspecDisturbances	character	land use category- specific disturbances, e.g. stock proof	``text''
PhysIndicators	character	physical indicators, indicating alteration of physical properties of site	``brn'',``bp'',``it",``am'',``po'',``du''
MacroHabitat	character	habitat classification after Fossitt, 2000	``GA1'',``GS3'',``GS4",``PB1'',``PB2'',``PB3'',``PB4",``PF1'',``WD4'',``WS5''
DrainIntensity	numeric	estimated frequency of drains on 1 ha area of site	num values
DrainDistance(m)	numeric	measured distance between drains	num values
DrainWaterDepth(cm)	numeric	measured water table depth below surface in drains close to sampling point	num values
AvgDrWatDepth	numeric	mean measured water table depth below surface in drains per site	num values
DrainStatus	character	status of drains	``fun'',``vegunbl'',``bl''
OtherFeatures	character	other features related to drainage and site improvement properties	``bunds'',``dams''
ActualMoisture	character	qualitative measure of current surface moisture on site per point	``rd'',``mw'',``wtw",``pp'',``swa''
pHSurWat	numeric	measured acidity of surface water bodies present on site	num values
ECSurWat(mS/cm)	numeric	measured actual electric conductivity of surface water present on site in micro-Siemens per centimeter (mS*cm^−1)	num values
TempSurWat(°C)	numeric	measured temperature of surface water present on site in degree Celsius (°C)	num values
AvgpHSurWat	numeric	mean measured acidity of surface water present on site (3 measurements)	num values
AvgECSurWat(mS/cm)	numeric	mean measured actual electric conductivity of surface water present on site in micro-Siemens per centimeter (mS*cm^−1) (3 measurements)	num values
ForRotation	numeric	time of forestry rotation on site	num values
ForAgeStand(yr)	numeric	age of forest stand on site in years (yr)	years
ForPlantingDistance(m)	character	estimated planting distance of trees in a forestry plantation	``2 × 2″
ForSpecies	character	species of forest stand	``Picea abies'',``Picea sitchensis'',``Pinus contorta''
AddRemarks	character	additional site-specific remarks	``text''
DepthAvg(cm)	numeric	average depth of sampling points per site in centimenter (cm)	num values
DepthSD(cm)	numeric	standard deviation of depth of sampling points per site in centimeter (cm)	num values
DistanceToDrain(m)	numeric	distance between sampling point and nearest drain in meter (m)	num values
MicroHabitat	character	micro habitat within a radius of 5 m around sampling point	``pat'',``hum'',``hol",``sphhum'',``pls'',``sphcusp",``lws'',``fl'',``flt'',``lk'',``sk",``rv'',``bp'',``ts'',``md''
MicroTopo	character	micro topography within a radius of 5 m around sampling point	``fl'',``gsl'',``gund'',``sund'',``dep",``ow'',``hum''
SamplingDate	character	date of sampling	``dates''
addRemarks	character	additional point-specifc remarks	``text''
Cov_*Sphagnum*(%)	numeric	cover of *Sphagnum* spp. mosses in percent (%)	num values
Cov_OtherMosses(%)	numeric	cover of non-*Sphagnum* spp. mosses in percent (%)	num values
Cov_Lichens(%)	numeric	cover of lichen spp. in percent (%)	num values
Cov_Forbs(%)	numeric	cover of herbaceous flowering plant spp. -excluding graminoids- in percent (%)	num values
Cov_EricoidShrubs(%)	numeric	cover of Ericaceae spp. vegetation in percent (%)	num values
Cov_WoodyVegetation(%)	numeric	cover of non-Ericaceae spp. type shrubs and bushes and small trees in percent (%)	num values
Cov_SedgesRushes(%)	numeric	cover of Cyperaceae spp. and Juncaceae spp. in percent (%)	num values
Cov_Grasses(%)	numeric	cover of Poaceae spp. Vegetation in percent (%)	num values
Cov_PlantLitter(%)	numeric	cover of plant litter -excluding senescent and dead plant parts- in percent (%)	num values
Cov_PlantLitterTot(%)	numeric	cover of total plant litter -including senesscent and dead plant parts- in percent (%)	num values
Cov_BarePeat(%)	numeric	cover of bare peat without cover or mat in percent (%)	num values
Cov_BarePeatAlgalMat(%)	numeric	cover of bare peat with algal mat in percent (%)	num values
Spec_*Sphagnum*	character	genus/species of *Sphagnum* mosses	``char values''
Spec_OtherMosses	character	family/genus/species of non-*Sphagnum* mosses	``char values''
Spec_Lichens	character	family/genus/species of lichens	``char values''
Spec_Forbs	character	family/genus/species of herbaceous flowering plants -excluding graminoids	``char values''
Spec_EricoidShrubs	character	family/genus/species of Ericaceae vegetation	``char values''
Spec_WoodyVegetation	character	family/genus/species of non-Ericaceae-type shrubs and bushes and small trees	``char values''
Spec_SedgesRushes	character	family/genus/species of Cyperaceae and Juncaceae vegetation	``char values''
Spec_Grasses	character	family/genus/species of Poaceae vegetation	``char values''
Spec_PlantLitter	character	family/genus/species of plant litter	``char values''
Spec_BarePeat	character	peat type on surface of bare peat	``char values''
Spec_BarePeatAlgalMat	character	family/genus/species of algae	``char values''
BagID	character	unique identifier of sample bag	``char values''
Depth(cm)	character	intervals of sampling depth in cm intervals (cm)	``0–10”,``10–25”,``25–50”,“50–75”,“75–100”,“100–150”,“150–200”,“200–250”,“250–300”,“300–350”,“350–400”,“400–450”,“450–500”,“500–550”,“550–600”,“600–650”,“650–700”,“700–750”,“750–800”,“800–850”,“850–900''
Volume(cm-3)	numeric	sample volume in cubic centimeter (cm^3)	20…884
Water(mass%)	numeric	gravimetric sample water content in percent of wet sample mass (mass%)	num values
Water(vol%)	numeric	volumetric sample water content in percent volume of sample volume (vol%)	num values
BD(g/cm-3)	numeric	bulk density in gramm per cubic centimeter dry mass (g*cm^−3)	num values
pHh2o	numeric	acidity of sample in water (H2O)	num values
EC(mS/cm)	numeric	Electrical conductivity of sample in sludge in micro Siemens / centimeter (mS*cm^−1)	num values
Temp( °C)	numeric	temperature of sludge for measuring electric conductivity	num values
VonPost	integer	von Post degree of decomposition on a scale of H1 to H10 (1 to 10)	1:10
Characteristics	character	qualitative assessment of peat substrate characteristics	``text''
PeatType	character	qualitative assessment of peat substrate type	``char values''
SampleMoisture	integer	qualitative assessment of sample moisture in parallel with Von post assessm,e	1:5
BasalPeat	character	type of basal peat	``bog'',``fen''
SubpeatSubstrate	character	substrate of sub-peat mineral layer below sedentary peat accumulation	``char values''
OM(mass%)	numeric	organic matter of sample in percent of dry mass (mass%) (duplicate mean)	num values
Ash(mass%)	numeric	ash of sample in percent of dry mass (mass%) (duplicate mean)	num values
SD(mass%)	numeric	standard deviation of organic matter and ash in percent of dry mass (mass%)	num values
CHNSID	integer	Unique identifier for bulk samples of CHNS analysis	num values

A third worksheet, ‘PeatCHNSO’, contains data from the elemental analysis of 291 composite peat samples (three observations are from redundant samples and are marked with a minus sign (-) in front of the CHNSID). The fourth worksheet ’MetadataPeatCHNSO’ describes 25 variables of this dataset following the same the descriptors (Table 2).

**Table 2:** Metadata of the worksheet ‘PeatCHNSO’ with 25 variables. The column with full names of acronyms is included in the database and not shown here; *ColumnName* contains the variable names.

ColumnName	VariableType	Description	Ranges or Acronyms
CHNSID	integer	unique identifier of CHNS sample	1:288
SampleName	character	name of sample	``text''
CHNSdepth	numeric	depth level of bulk sample	1:6
SiteID	integer	unique identifier of sampling site	1:50
SiteName	character	name of sampling site	``text''
LUC	character	land use category	``nat'',``for'',``gra'',``extdom'',``extind''
Management	character	management	``undr'',``dr'',``rw''
Moisture Content (% Wet Basis)	numeric	moisture content of sample on percent of wet basis (% Wet Basis)	num values
Ash (%DryMass)	numeric	ash content of sample in percent dry mass (%DryMass	num values
Carbon (%DryMass)	numeric	carbon content of sample in percent dry mass (%DryMass)	num values
Hydrogen (%DryMass)	numeric	hydrogen content of sample in percent dry mass (%DryMass)	num values
Nitrogen (%DryMass)	numeric	nitrogen content of sample in percent dry mass (%DryMass)	num values
Sulphur (%DryMass)	numeric	sulphur content of sample in percent dry mass (%DryMass)	num values
Oxygen (%DryMass)	numeric	oxygen content of sample in percent dry mass (%DryMass)-by difference	num values
Ash (%WetMass)	numeric	ash content of sample in percent wet mass (%WetMass)	num values
Carbon (%WetMass)	numeric	carbon content of sample in percent wet mass (%WetMass)	num values
Hydrogen (%WetMass)	numeric	hydrogen content of sample in percent wet mass (%WetMass)	num values
Nitrogen (%WetMass)	numeric	nitrogen content of sample in percent wet mass (%WetMass)	num values
Sulphur (%WetMass)	numeric	sulphur content of sample in percent wet mass (%WetMass)	num values
Oxygen (%WetMass)	numeric	oxygen content of sample in percent wet mass (%WetMass)-by difference	num values
Carbon (%DAF)	numeric	carbon content of sample in percent ash-free basis (%DAF)	num values
Hydrogen (%DAF)	numeric	hydrogen content of sample in percent ash-free basis (%DAF)	num values
Nitrogen (%DAF)	numeric	nitrogen content of sample in percent ash-free basis (%DAF)	num values
Sulphur (%DAF)	numeric	sulphur content of sample in percent ash-free basis (%DAF)	num values
Oxygen (%DAF)	numeric	oxygen content of sample in percent ash-free basis (%DAF)-by difference	num values

An R script was developed and executed to merge separate datasets (.csv) for each sampled site into two large Excel (.xlsx) files, resulting in worksheets ‘PeatlandProperties’ and ‘PeatCHNSO’ [8].

### 3.3 Water table monitoring datasets

The water table monitoring datasets (.xlsx) of the 8 monitored peatland sites are named according to their Land Use Category (e.g., “Nat” = Near-intact and undrained, with absent land use and management, ”For” = Forestry, “Extdom” = Domestic Extraction, “Gra” = Grassland), their site name (i.e., “Shk” = Sheskin, “Scb” = Scohaboy) and a differentiator that specifies the dataset as water table monitoring dataset (i.e. “WT”).

Each Excel-file is equipped with two worksheets. The first, using the same name as the file itself, contains monitoring data. The first row specifies the variables, including Site-ID, Date and Time and the names of the points where water table was monitored. Variables (or columns) 3 to 6 (‘WT_Orpheus’, ‘Ref_Orpheus’, WTOrpheus_Adj’) show the continuous measurements of the OTT-Orpheus Mini® water logger in a 60-minute interval. All subsequent variables describe randomly allocated measurement points, either located at peat sampling points (in this case the peat sampling point number is specified in ‘metadata’), or in microhabitats across monitoring sites (name of microhabitat or location is given).

The ‘metadata’ worksheet specifies the coordinates of the monitoring sites and the names of each variable where water level was monitored as well as the Peatland Type-Land Use-Management category that is used in the AUGER Peatland Data database. Each of the eight datasets contains a report of one coordinate in the metadata (Table 3).

**Table 3:** An example of the metadata for water table monitoring sites (here RB-nat-undrained: undrained, near-intact raised bog), describing variables of each monitoring site. Coordinates (X, Y) are given for each monitoring site in decimal degrees.

Variable	Description	Coordinates	
SiteID	Site	52.9831475	−8.0519271
Date	Date		
Time	Time	AOD (m)	74.975
WT_Orpheus	measurement at logger		
Ref_Orpheus	reference at logger		
WTOrpheus_Adj	measurement minus reference at logger		
WT_col_258	measurement at point 258		
Ref_col_258	reference at point 258		
WT_col_258_Adj	measurement minus reference at point 258		
WT_drw	measurement at ditch-rewetted		
Ref_drw	reference at ditch-rewetted		
WT_drw_Adj	measurement minus reference at ditch-rewetted		
WT_veg_259	measurement at point 259		
Ref_veg_259	reference at point 259		
WT_veg_259_Adj	measurement minus reference at point 259		
WT_pool_260	measurement at point 260		
Ref_pool_260	reference at point 260		
WT_pool_260_Adj	measurement minus reference at point 260		
WT_hum-veg	measruement at hummock-vegetated		
Ref_hum-veg	reference at hummock-vegetated		
WT_hum-veg_Adj	measurement minus reference at hummock-vegetated		
SiteName	Scohaboy		
PLT-LUC-Mgmt	RB-nat-undrained		

### 3.4 Data usage

Datasets are arranged in a way that navigation is intuitive. The first row and first column are fixed due to the long and wide format of the datasets. This aids users in scrolling to the end of the dataset without losing oversight of the sample ID (first column) or variable name (first row).

Typical workflows include converting the Excel file to a .csv-file to import in R or other statistical software for further analysis or visualisation. Each column (variable) allows for (sequential) filtering, to support exporting a specific set of data only, if required.

The metadata worksheets help guide users to navigate the peatland database and water monitoring datasets. They specifically aid in explaining the different variables of the ‘PeatlandProperties’ and ‘PeatCHNSO’ worksheets, and different water monitoring datasets. Each variable (*ColumnName*) is listed in a separate row, explaining its variable type (*VariableType*) describing the variable (*Description*), their specification either as a range (for integer variables), numeric values (for numeric variables), or acronym (for levels of categorial variables). A last column provides the full names of the variable levels (*FullNames* – visible only in the publicly available peatland database metadata).

Categorial variables were coded using predefined controlled vocabularies derived from a fixed list of terms and were applied consistently throughout the datasets. Variable types and their numeric ranges or levels are given with a description in the respective metadata tables of the datasets.

All cells in the datasets without a value (empty cells) are equivalent to ‘NA’. These indicate that no value from the defined list of acronyms for levels could be defined for the respective variable, or that no numerical value was measured, either because there simply was no presence or the variable could not be measured during sampling. Cells with the numerical value 0 contain the number 0.

## 4 Experimental Design, Materials and Methods

### 4.1 Peatland survey

**Fig. 1:**
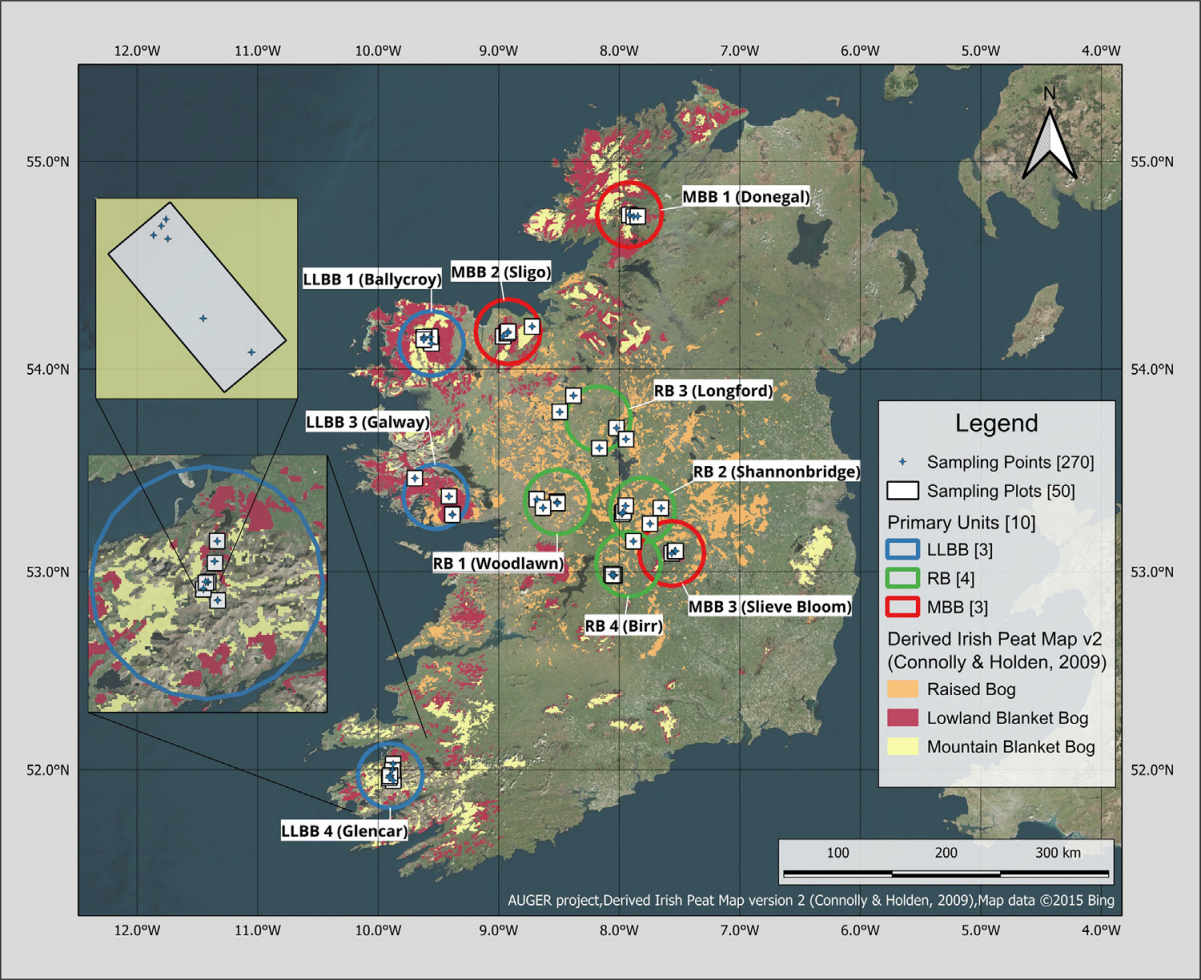
Map of Ireland showing location of the sampling units in the study. Sampling points (blue stars) were selected within sampling plots (white quadrats) which were nested within primary units (coloured circles). Peat coverage was adapted from [3]; Map created using Ordnance Survey Ireland data and QGIS. Base map: Bing.

A peatland sampling campaign was conducted across the Republic of Ireland between May 2017 and July 2019 as part of the AUGER project, funded by the Irish Environmental Protection Agency [8]. Data collection followed a multi-stage sampling design, with vegetation assessments by 1 m^2^ relevées, peat sampling using a Russian peat sampler, and visual description and validation of land use, management, macro-and microhabitats and landscape site features. 50 plots were sampled with a total of 4024 soil samples from 270 sampling points. Peat samples were used for subsequent peat soil analyses (Fig. 1).

Vegetation was assessed before sampling the peat soil using 1 × 1 m relevées. Assessments was determined on the level of plant functional types (PFT: *sphagnum mosses, other mosses, lichens, forbs, ericoid dwarf shrubs, woody vegetation, sedges & rushes, grasses, plantation trees (coniferous, deciduous, mixed), plant litter, bare peat, bare peat with algal mat*). PFT cover was estimated in steps using the nearest 3% for cover below 10%, and the nearest 5% from a cover of 10% onward. The main genus of each PFT present was recorded, where possible down to the species level. Microhabitat and microtopography were assessed within a 5-meter radius around the vegetation relevées.

Peat sampling was conducted along the entire depth of the peat substrate with fixed depth intervals (0–10; 10–25; 25–50; 50–100; 100 cm for depths ≥ 100 cm) and subsampling was conducted on each peat sample to analyse 15 edaphic peatland properties. Living plant biomass was removed before augering to ensure exclusion from the soil sample by carefully separating the living biomass from the non-living peat plant mass of the peat substrate. Peat depth was measured at three augering points of each sampling point (n = 3) using a Russian auger and averaged to one depth value (arithmetic mean). Where necessary, a 5 mm diameter fibreglass rod was used. The visible boundary between the deepest peat sample and the mineral substrate below in the deepest extracted peat core was taken as boundary. Where marl sediment was present (mainly below raised bogs), the point of the start of colour change (dark brown to whitish yellow) served as the boundary. Where hard bedrock was encountered below the blanket bog types, peat depth was measured as the cumulative length to the last core plus 10 cm for the steel tip of the Russian auger.

Peat type was determined on plant group level, with the dominant groups identified in the order of their quantitative dominance in the peat substrate (i.e. ‘Sphagnum-Sedge’, or Sedge-Sphagnum-Wood). The analysis only provides information on the peat vegetation 'mix' in the substrate but not on the exact quantities or proportions of the different plant groups in the peat substrate.

A water table monitoring campaign was conducted between November 2017 to January 2020, providing continuous measurement records of water levels at eight peatland sites (measurement frequency: 60-minute interval) using water level loggers (OTT Orpheus Mini®) installed in fixed PVC columns (Ø 30 cm), and were supported by manual measurements using a water level probe.

### 4.2 Survey sampling design

**Fig. 2:**
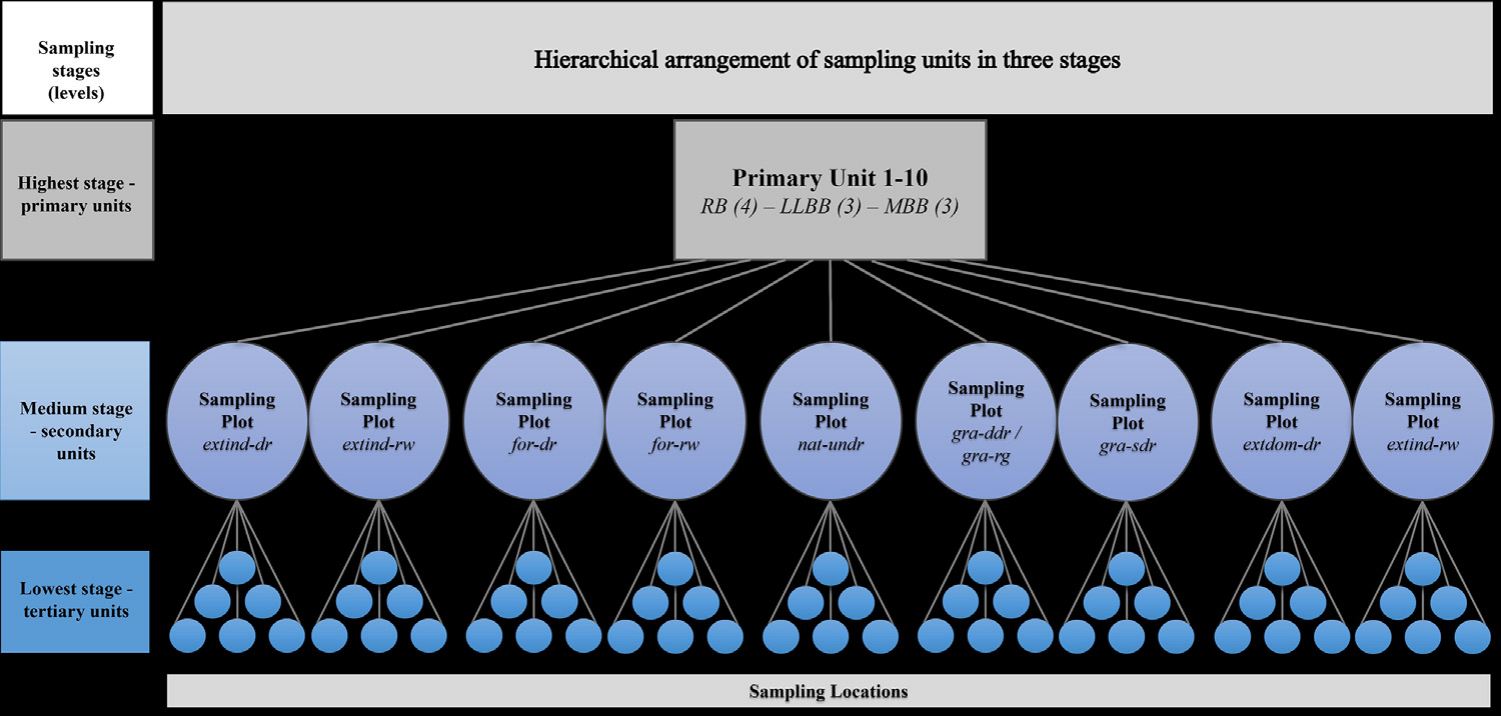
Multistage-stratified sampling design with hierarchical arrangement of sampling units in three stages. Sampling units of lower stages were nested within units of higher stages. Strata are denoted by *italic* text within each box, e.g.*RB-extind-dr*. Note that not all strata existed within primary units. The graphic shows all theoretical strata and sampling plots, out of which a varying number was sampled within each primary unit.

The data was collected in a multi-stage stratified sampling design. Sampling units were randomly selected for each of the three stages from an assumed infinite, stratified population of all ombrotrophic peatlands in Ireland. Soil samples were taken on sampling units arranged in a nested design (Fig. 2), where sample sizes differed for each stage (Table 4)*.* At the lowest stage, 270 sampling points (or locations) (SL - *level 1*) were nested within a higher stage of 50 sampling plots (SP - *level 2*) which was again nested within the highest stage of 10 primary units (PU - *level 3*) (Table 5). Circular-shaped primary units (radius = ∼30 km) were randomly selected within the three peatland type strata RB, LLBB, MBB (sample sizes n_PU_ = 4, 3, 3). One sampling plot was then randomly selected at each site within a specific land use-management stratum (LUC:Mgmt), which was repeated for each primary unit. Sampling plots were rectangular in shape with an area of ∼1 ha. Care was given to include sampling plots that were located at the edges of the peatlands in the case of domestic extraction land use plots. As not every land use was present within each identified primary unit (e.g. no industrial peat extraction in mountain blanket bogs), sample sizes varied between primary units (n_SP_ = 4–5). Finally, a simple random sample of sampling points was selected within each sampling plot and sample sizes were calculated for each sampling plot individually using a cost-constrain function [9]. The exact location of the vegetation relevée and peat soil sampling cores was then determined, equally representing the microhabitats (e.g. hummocks, hollows) present in a 5-meter radius around each sampling point.

**Table 4:** Sample sizes of peatland survey for each type of sampling unit. Three stages were devised within a hierarchical sampling design.

Sampling unit	Sample Size
Primary units	10
Secondary units (Sampling plots)	50
Tertiary units (Sampling points)	270

**Table 5:** Details of sampling support for measured/assessed peatland properties and sampling site/point characteristics with sample sizes. Sampling support varied for each assessed peatland property.

Peatland property	Sampling support	Sample size
Vegetation	Relevée 1 × 1 m^2^	270
Soil	‘Russian’ peat auger cores, half-cylindrical form, aliquots with varying volumes btw. 20 - 500 cm^3^	2012
Sampling site characteristics	Polygon of random size	50
Sampling point characteristics	Circle, 5 m radius around sampling point	270

The hierarchical structure of the sampling design accounts for potential autocorrelation and addresses the issue of dependency of sampling units in geographical space [10]. A classification of sampling design classes and a decision tree was followed [11].

### 4.3 Primary units (sampling areas)

**Fig. 3:**
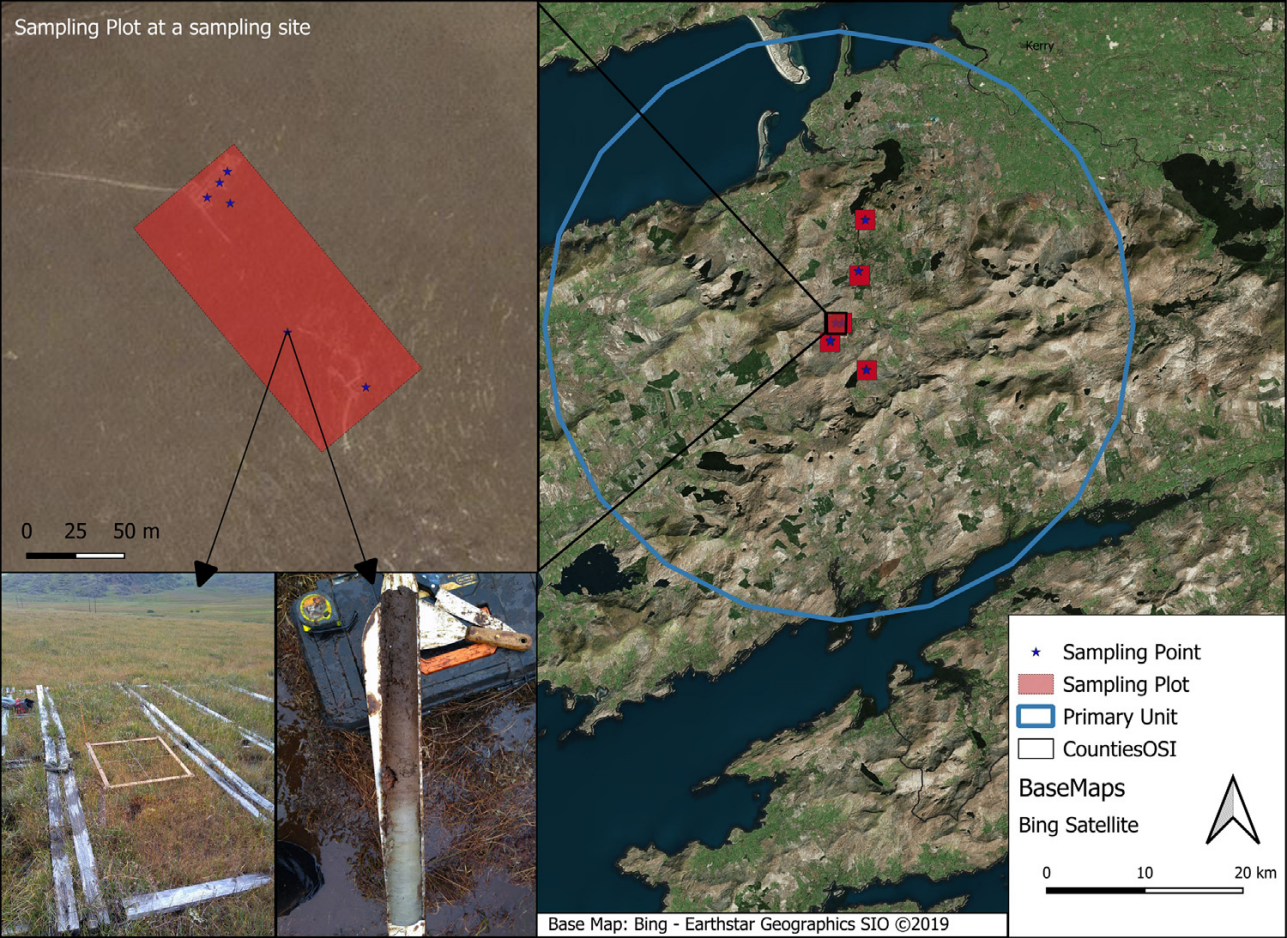
Graphic depicting the three different stages of sampling with units of the survey. Sampling locations were nested within sampling plots-randomly sampled at sampling sites (left side); plots were nested within primary units (right side), randomly sampled across three main peatland types; the peat core illustrates the colour change between organic peat and mineral subtrate of a clay sediment underlying the peat, used to confine total peat depth.

Primary units were selected based on identified areas of a remote sensing exercise using QGIS. Points were allocated to areas where all strata were represented. Previous research sites, for which legacy data on peatland properties already existed, were given priority over new sites. A supplementary objective of the survey was to complement existing greenhouse gas and vegetation data from previous research sites with peat soil data. At the same time, the primary units had to equally represent the three major ombrotrophic peatland types RB, LLBB and MBB. Finally, 30 km buffer zones were constructed around the selected points (Fig. 3).

### 4.4 Secondary units (sampling plots)

Sampling plots were selected in a stratified simple random sampling approach for each single stratum. Several random points were allocated to each stratum using QGIS within the boundaries of each primary unit. These random points represented the centers of potential sampling plots with size of ∼1 ha. A sequence of selection criteria was followed, with priority to select sites with existing legacy data. Aspects of site accessibility and trafficability as well as minimized travel costs (travel time) were also considered to choose the best accessible sampling plots. Eventually, one sampling plot per stratum was randomly selected.

### 4.5 Tertiary units (sampling points)

A maximum of six sampling points (n ≤ 6) were selected within each sampling plot as simple random sample. Selection was based on a collection of pre-assigned random points in QGIS, but decision on sampling sizes for each sampling plot was dependent on average depths of sampling plots, measured at each sampling point. Practically, sampling sizes were limited by time constraints during fieldwork and the large number of resulting peat samples.

**Fig. 4:**
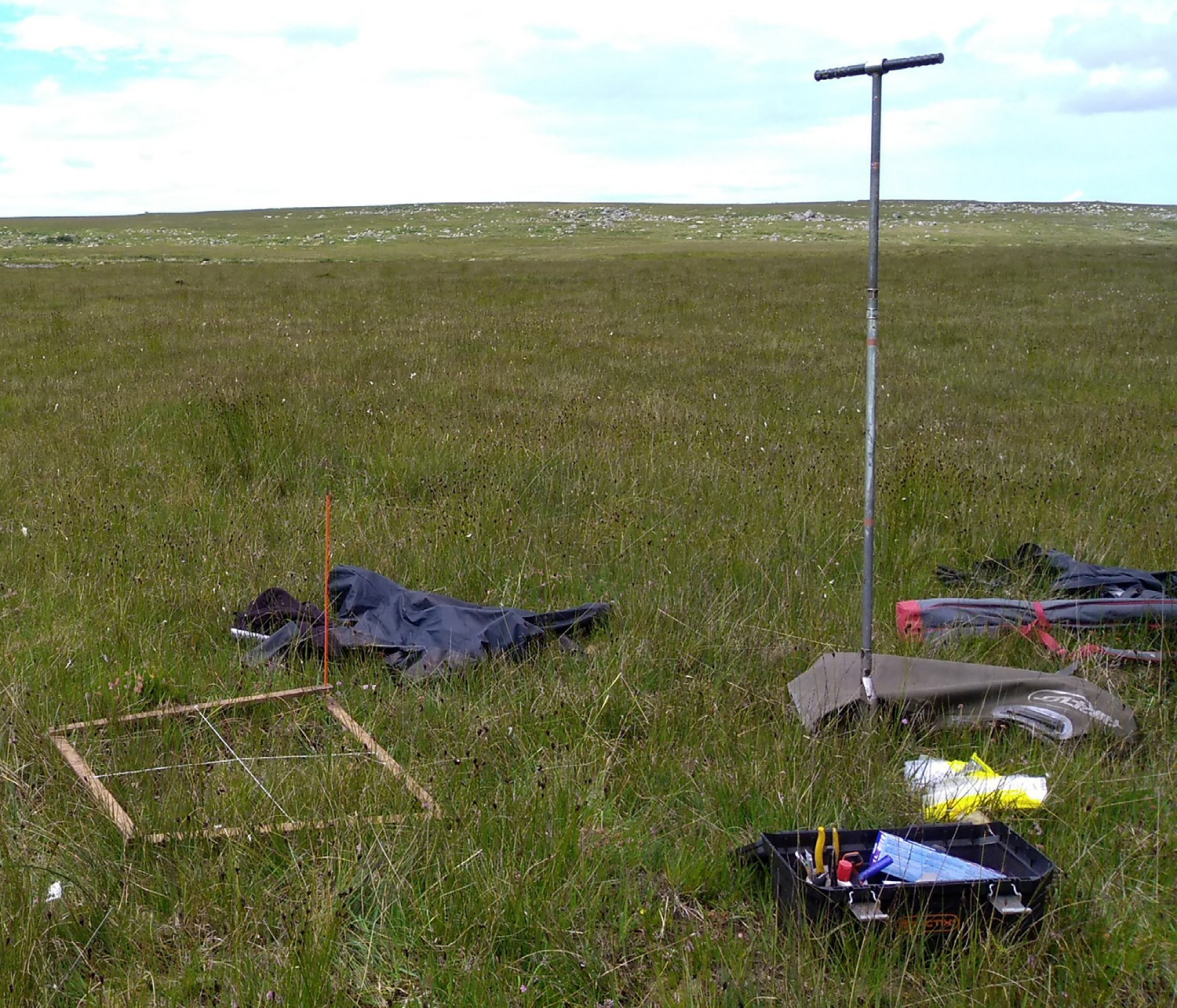
Sampling equipment used for the peatland survey, including a quadratic relevée (bottom left), an orange-coloured 5 mm fibreglass-rod for measuring peat depth (centre left), a ‘Russian’ peat sampler (centre right), a toolbox with sample bags (bottom centre) and large bags for carrying samples and sampling equipment (centre right). Photograph were taken by the first author in County Galway, June 2018.

**Fig. 5:**
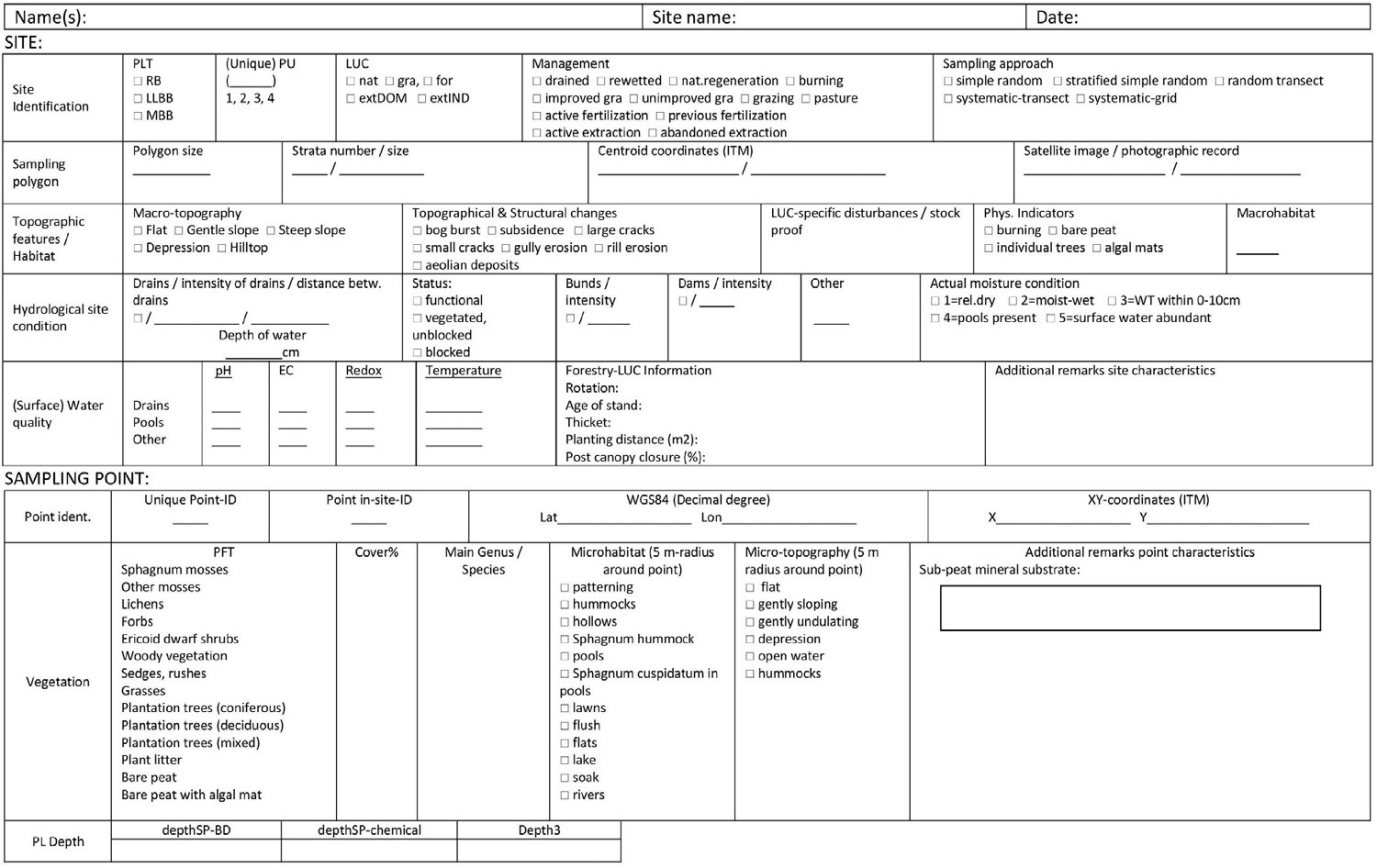
Field sampling sheet for recording sampling site-specific, location-specific characteristics, vegetation assessment data, depths at sampling locations and other information.

Soil sampling was conducted within each sampling plot and sampling points were used for extracting two types of samples from fixed, consecutive depths, with help of a ‘Russian’ peat sampler (Fig. 4). In addition, data was collected about sampling site-specific and sampling point-specific characteristics. Site-specific characteristics were assessed within areas of sampling site boundaries, defined by the land use- management of the respective sampling site. Location-specific characteristics were assessed within a 5-meter radius around a sampling point (Table 5). A field sampling sheet was designed and used for data collection during the survey (Fig. 5).

Various layers of raster and shape files with soil, peatland and land cover data were the basis for the selection of sampling units (Table 6). Using QGIS, files were combined to generate a one-layer file of intersected layers from the raster and shape files. This file served as overlay for base maps to select primary units in a remote sensing exercise and formed the basis for stratification. In addition, a list with all potential sampling sites was compiled, including sites where previous research has been conducted.

**Table 6:** Specifications of datasets used for selection of sampling units.

Dataset	Type / Format of data	Author(s) / Access link
Derived Irish Peat Map v2 (DIPMV2)	areal extent of peatlands-national scale raster & shape format	Correspondence with author [3]
CORINE land cover map 2012	land cover classes for Ireland shape format	K. Lydon, G. Smith, CORINE Landcover 2012 Ireland
Irish Soil Information System (ISIS)	soil series and associations of Ireland shape format	Teagasc, EPA, C. University, Irish Soil Information System, (2015). http://gis.teagasc.ie/soils/downloads.php
Soil and Subsoils Mapping Project	soil and subsoils Ireland shape format	Teagasc-EPA Soil and Subsoil Mapping Project
Coillte Forest cover map	forestry polygons with information on plot and species	Correspondence with staff in Coillte
Base Maps for QGIS	satellite imagery Ireland raster format	QGIS QuickMap Services plugin

### 4.6 Laboratory methods

Soil samples were dried in a drying oven at 105 °C for at least 24 h and until a constant mass was reached after which sample weights were recorded. Gravimetric water content (*WC_grav_* in % wet soil) was measured from weight loss of samples before and after drying. Dry bulk density (*ρ_b_* in g cm^-3^) was calculated as the ratio between dry soil mass M_sd_ and the volume of the fresh soil sample V_sw_ [12]:

(1) $$r_{b}=\;M_{sd}/V_{sw},$$

Particle density *ρ_s_* was calculated using a formula from [13], with average OM-particle density values (1.44 g cm^-3^) for bog peat and floating sedge mat peat of Canadian ombrotrophic peatlands (Table 2, in [13]). Particle density values for the mineral fraction (2.65 g cm^-3^) were taken from [14]:

(2) $$\rho_{s}=\frac{\left(OM*1.44\;+\;Ash*2.65\right)}{100},$$

Porosity (Φ) was calculated from bulk density and particle density using the formula from [13]:

(3) $$\Phi =\;1-\;\rho_{b}/\rho_{s}$$

After drying, samples were milled to a grain size of 200 μm in a rotor mill (Fritsch Pulverisette 14®). Organic matter (*OM* in % dry matter) and ash-content (*Ash* in % dry matter) were measured on subsamples in a muffle furnace (Nabertherm B180 ®). Subsamples were corrected for water content at 105 °C overnight before measurement [15,16], and were manually homogenised with a spatula afterwards.

For measurements of elemental composition, soil samples that belonged to the same sampling plot were subsampled and bulked to form a composite sample across each specified depth interval for depths <50 cm. For depths >50 cm, one composite sample was formed from the combined depth intervals. An equal amount of subsample (1 g) was taken from each soil sample to form the composite sample across each depth interval; for the deepest samples with differing volumes at each sampling point, a proportional volume of subsample was taken. The composite samples represented weighted averages of the individual peat soil samples with an equal proportion of each individual sample. A detailed description of the protocol elaborated for forming the composite samples can be found in [17].

Total carbon (*C_tot_*), nitrogen (*N_tot_*), hydrogen (*H_tot_*), sulfur (*S_tot_*) and oxygen (*O_tot_*) were measured for each composite sample (% dry matter). Analyses were conducted in duplicate using an *Elementar Vario MACRO—Cube®* elemental analyser*.* Sample mass was corrected for water content by drying overnight [18]. The C:N ratio was calculated as the ratio between total C (*C_tot_*) and total N (*N_tot_*) contents:

(4) $$C:N=\;C_{tot}/N_{tot}$$

The pH measurements (*pH_H2O_*) were conducted on peat sludges of fresh semi-disturbed samples within 1–2 days after sampling. Electrical conductivity (*EC*) was measured after 20 h in the same peat sludges, using an EC meter (*WTW© Multi 9310 IDS*). A linear temperature compensation factor of 0.02 °C^-1^ (Riley 1986) was used to standardise measurements to EC_25_ (at 25 °C). Degree of peat decomposition (DD) was measured using the 10-point von Post scale, which ranges from H1 (completely undecomposed) to H10 (completely decomposed) [19].

### 4.7 Water table monitoring

**Fig. 6:**
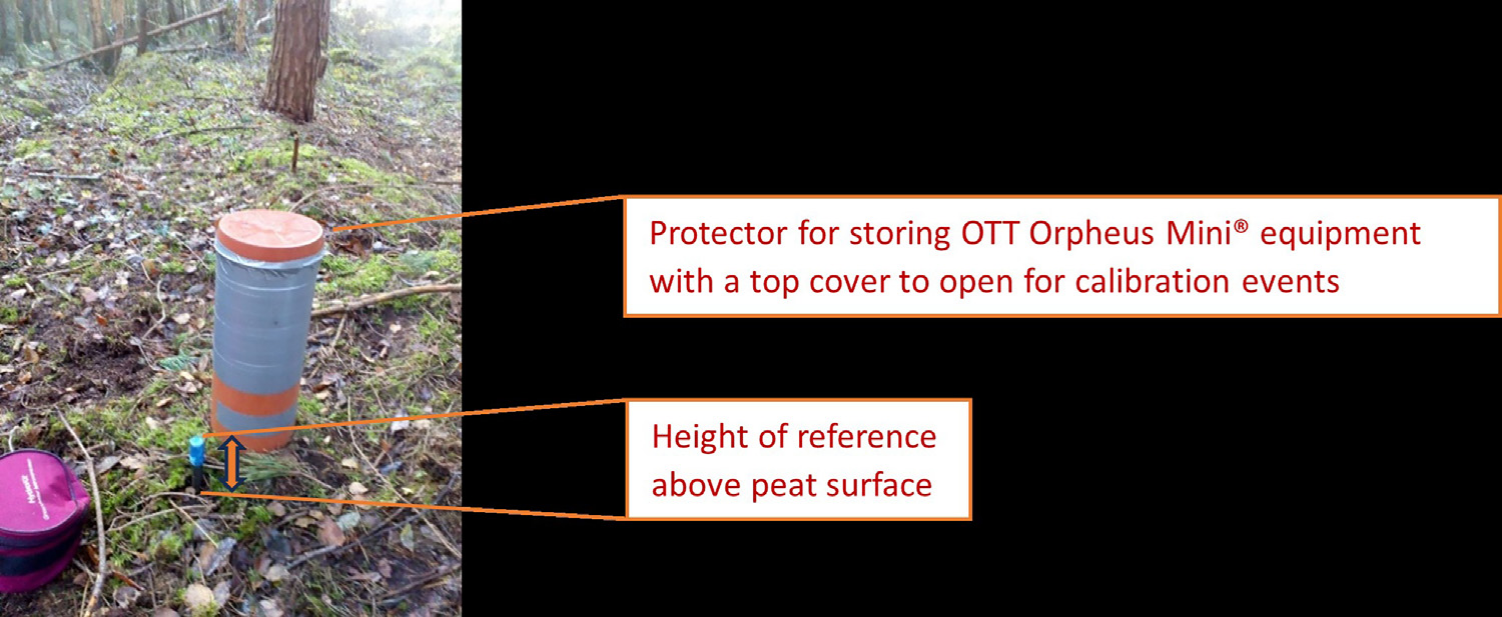
Installed water table measurement equipment. Water table measurement column (PVC, ∼30 cm diameter) with one of several hollow iron rods next to the 5 mm-wide PVC column (not visible in this picture as hidden below the orange protector).

Water table monitoring was conducted at eight sites of the peatland survey (Table 7). At two peatland sites (one raised bog and one lowland blanket bog site), 5 cm diameter PVC-columns were inserted at four Land Use Categories (LUC). An orange top cover was imposed onto the inserted column to protect it from weathering. One OTT-Orpheus Mini® WT-logger was installed within each PVC column (Fig. 6).

**Table 7:** Sites selected for hydrological monitoring for the period November 2017 to December 2019. Eight high-frequency loggers (OTT-Orpheus Mini®) were installed at eight sampling sites, covering four LUC at two peatland types, each. The name of each dataset is given in brackets, the term ‘Natural’ here is used only for naming purposes but does not reflect the actual state of the peatland site.

Site Names	Lat	Lon	X (Easting)	Y (Northing)	Elevation (m AOD)*
Knockmoyle-Sheskin (Co. Mayo) – Peatland Type: Lowland Blanket Bog (LLBB)					
LLBB-Natural (*NatShk_WT*)	54.1564535	−9.569419	97,533	324,173	
LLBB-Forest-drained (*ForShk_WT*)	54.1545388	−9.6212393	94,144	324,036	
LLBB-Cutover-drained (*ExtdomShk_WT*)	54.1547273	−9.6188683	94,299	324,054	
LLBB-Grassland-drained (*GraShk_WT*)	54.1427176	−9.6216846	94,085	322,721	
Scohaboy (Co. Offaly) – Peatland Type: Raised Bog (RB)					
RB-Natural (*NatScb_WT*)	52.9831475	−8.0519271	196,621	192,450	74.975
RB-Forestry-rewetted (*ForrwScb_WT*)	52.9835996	−8.0480808	196,820	192,512	74.020
RB-Forestry-drained (*ForScb_WT*)	52.984449	−8.0392027	196,824	192,490	71.931
RB-Cutover (*ExtdomScb_WT*)	52.9796974	−8.0468698	196,902	192,069	73.366

⁎ A differential GPS (Trimble®, accuracy of ± 0.05 m) was used to measure absolute levels above ordnance datum (m AOD) for Raised Bog sites (Scohaboy), which was not possible for the Lowland Blanket Bog sites (Knockmoyle-Sheskin) due to Covid-19 outbreak.

Water table measurements were continuously measured within a 60-minute interval. Each 4–6 weeks, the measurement devices were calibrated to ensure accurate measurement. Depth of water table was measured to a reference point (the tip of the 5 cm-wide, inserted PVC column) and then related to this reference height above peat surface.

In addition to the continuous measurements, water table was measured manually across each monitoring site during each calibration event of the OTT-Orpheus Mini water loggers (approx. every 4–6 weeks), from 3 cm-wide, hollow iron rods (2.4 cm internal diameter) that were inserted into the peat substrate at the start of the monitoring period (November 2017). A manual water depth probe connected to a measurement tape was inserted into the iron tubes (red bag in Fig. 6) to measure the depth of the water table below the peat surface.

## Limitations

Some sampling points were not accessible due to limited trafficability and site access. Alternative sampling points were used. At a few sampling points, hard wood in deeper peat layers obstructed further sampling, influencing measurement of peat properties and total peat depth, whereby sampling in such situations was not always successful at nearby sampling points. The sampling campaign was constrained by time due to the larger amount of sampling points, the distances between the sampling sites and the requirement of national sampling coverage.

The goal of the survey was to establish a peatland property dataset which is representative for the national level. A main objective was to explain variability across peatland types, land use categories and management types. Therefore, a clustered, multi-level sampling design was chosen which optimized the estimation accuracy on the national level while minimizing sample sizes on the peatland site level. The trade-off of this approach is that site-specific variability could only be explained at randomly allocated sampling plots (∼1 ha size), without fully explaining spatial variability across the larger area of the entire sampling site (e.g. a raised bog, or spatially delineated blanket bog area). However, random allocation of sampling plots enabled unbiased estimation and thus was assumed to guarantee representativeness of peatland properties for a given sampling site. Thus, the datasets provide a good basis for representative estimations of peatland properties (vegetation, edaphic properties, etc.) on a national level for each combination of peatland type, land use category and management type, or combinations thereof. Hence, robust site-level estimations would require additional repetitions of sampling plots on the same peatland site.

Similarily, the peatland database does not provide repeated measurements of the same sampling points, sampling plots or primary units. All three levels were sampled only once, thus providing spatially representative data without information on temporal change of peatland properties. To this effect, the datasets have the character of reference or baseline data providing a good basis for future research on temporal change of peatland properties.

For some of the peat properties, sample sizes are relatively small causing large margins of error for peat property estimates. A trade-off between increased sample sizes and sampling depths needed to be levelled out when designing the peatland survey, with different peat properties having different variability. Due to financial and logistical constraints, it was not possible to account for varying sample sizes per peat property.

The qualitative analysis of peat type only provides information about the peat vegetation in the substrate but does not give exact quantities or proportions of the different plant groups in the substrate (e.g. “*Sphagnum-*Sedge-woody peat”). The analysis involved a visual assessment without a quantitative determination of vegetation composition.

The water table dataset consists of a small number of monitored sites (n = 8), spreading over a 2-year period. This is a relatively short temporal coverage limiting the use of the data to analysis of inter-annual variation of water tables but preventing multi-annual assessments. Due to the missing repetitions of sites, this data should serve as a reference to more representative measurements including additional site repetitions.

## Ethics Statement

The authors have read and follow the ethical requirements for publication in Data in Brief and confirm that the current work does not involve human subjects, animal experiments, or any data collected from social media platforms.

## CRediT Author Statement

**Table:** 

Contributor role	Role definition	Name co-author
Conceptualization	Ideas; formulation or evolution of overarching research goals and aims.	Kilian Walz
Methodology	Ideas; formulation or evolution of overarching research goals and aims	Kilian Walz
Software	Programming, implementation of the computer code and supporting algorithms; testing of existing code components	Kilian Walz
Validation	Verification, whether as a part of the activity or separate, of the overall replication/reproducibility of results/experiments and other research outputs.	Kenneth A. Byrne, Florence Renou-Wilson
Formal analysis	Application of statistical, mathematical, computational, or other formal techniques to analyse or synthesize study data.	Kilian Walz
Investigation	Conducting a research and investigation process, specifically performing the experiments, or data/evidence collection.	Kilian Walz David Wilson
Resources	Provision of study materials, reagents, materials, patients, laboratory samples, animals, instrumentation, computing resources, or other analysis tools.	Kenneth A. Byrne
Data curation	Management activities to annotate (produce metadata), scrub data, and maintain research data (including software code, where it is necessary for interpreting the data itself) for initial use and later reuse.	Kilian Walz, Florence Renou-Wilson
Writing – original draft	Creation and/or presentation of the published work, specifically writing the initial draft (including substantive translation).	Kilian Walz
Writing – review & editing	Preparation, creation, and/or presentation of the published work by those from the original research group, specifically critical review, commentary or revision – including pre- or post-publication stages.	Florence Renou-Wilson, Kenneth A. Byrne
Visualization	Preparation, creation, and/or presentation of the published work, specifically visualization/data presentation.	Kilian Walz
Supervision	Oversight and leadership responsibility for the research activity planning and execution, including mentorship external to the core team.	Kenneth A. Byrne, Florence Renou-Wilson
Project administration	Management and coordination responsibility for the research activity planning and execution.	Kilian Walz, Ken Byrne, Florence Renou-Wilson
Funding acquisition	Acquisition of the financial support for the project leading to this publication.	Florence Renou-Wilson, Kenneth A. Byrne

## Data Availability

ZenodoA national peatland database for the Republic of Ireland (Original data)
